# 2NPLGBM: a genomic model that merges the strengths of classical and machine learning methods in genomic prediction

**DOI:** 10.1186/s13007-026-01545-2

**Published:** 2026-05-28

**Authors:** Bright Enogieru Osatohanmwen, Indalécio Cunha Vieira, Ahmad Reza Sharifi, Timothy M. Beissinger

**Affiliations:** 1 Division of Plant Breeding Methodology, Department of Crop Sciences, University of Goettingen, 37075 Goettingen, Germany; 2 Center for Integrated Breeding Research, University of Goettingen, 37075 Goettingen, Germany; 3 KWS SAAT SE & Co. KGaA, Einbeck, Germany; 4 Division of Animal Breeding and Genetics, Department of Animal Sciences, University of Gottingen, 37075 Goettingen, Germany; 5 Heritable Agriculture, San Carlos, California 94070 USA

**Keywords:** Genomic prediction, Hybrid breeding, LightGBM, Dominance, Non-additive effects, Machine learning, 2NP matrix, SHAP, Maize, Additive–dominance modeling, GBLUP, Selection efficiency, Temporal validation

## Abstract

**Background:**

Genomic prediction (GP) is a central component of modern plant breeding, enabling the early selection of superior genotypes based on genomic marker data. Classical GP models, such as genomic best linear unbiased prediction (GBLUP), operate within the data modeling culture and typically assume additive genetic effects, with extensions required to model non-additive effects such as dominance and epistasis. In contrast, machine learning (ML) models from the algorithmic modeling culture can flexibly model complex, non-additive genetic relationship but often lack direct grounding in quantitative genetic theory and interpretability. To bridge these gaps, we propose 2NPLGBM, a hybrid genomic prediction approach that integrates quantitative genetics with ML. This method introduces a two-matrix (2NP) genotype representation by concatenating additive (Z) and dominance (W) matrices, which are then used as input to a Light Gradient Boosting Machine (LGBM), enabling the simultaneous modeling of additive, dominance, and higher-order genetic interactions (AA, AD, DD).

**Results:**

The 2NPLGBM model was evaluated using six years of test-cross hybrid maize trial data across four agronomic traits (grain yield, plant height, days to silking, and days to anthesis) under five cross-validation schemes simulating temporal: Leave-One-Year-Out (LOYO), Rolling Window (RW), and genetic generalization: Five-Fold, and tester-based schemes (Tester CV0 and Tester CV00). Compared to GBLUP, 2NPLGBM achieved an average of 5% improvement in predictive accuracy under temporal validations and over 15% gains under tester-based schemes, particularly for flowering traits (days to silking and days to anthesis). Performance was generally comparable to LGBM, with both ML models outperforming GBLUP for most traits. Under Tester CV0, 2NPLGBM showed its strongest relative advantage over LGBM for flowering traits, suggesting improved capture of interaction-related genetic signals, whereas LGBM generally performed best for plant height and grain yield. In five-fold CV and Tester CV00, GBLUP remained competitive for some traits, while both machine learning models showed reduced gains, with LGBM slightly outperforming 2NPLGBM. In addition, 2NPLGBM generally improved selection efficiency over GBLUP and, in most cases, LGBM, indicating enhanced ability to capture complex genetic signals relevant for hybrid ranking, particularly for flowering traits, whereas LGBM tended to achieve the highest selection efficiency for plant height and grain yield. Feature interpretation using Shapley Additive exPlanations (SHAP) confirmed that non-additive interactions contributed substantially to prediction accuracy for highly heritable traits. It also revealed trait-specific architectures, additive effects dominated flowering traits, while dominance effects contributed more to plant height and yield. Classical variance component analysis supported these findings, indicating high dominance contributions of 17.3% for yield and 8.2% for plant height.

**Conclusion:**

The 2NPLGBM model integrates quantitative genetic theory with machine-learning, bridging classical statistical (data-model) and algorithmic modeling cultures. bridging classical statistical (data model) and algorithmic modeling cultures. By jointly modeling additive and non-additive effects it can improve predictive accuracy, interpretability, and selection efficiency in test-cross hybrids. Future work should explore multi-trait and multi-environment extensions, integration of environmental covariates, and the inclusion of multi-omic data to further strengthen predictive power and interpretability.

**Supplementary Information:**

The online version contains supplementary material available at 10.1186/s13007-026-01545-2.

## Introduction

Plant breeding has advanced significantly with the introduction of genomic selection (GS). This methodology leverages genome-wide molecular markers to predict the genetic potential of individuals within a breeding population. This process, known as genomic prediction, enables breeders to estimate breeding values based on dense marker data, allowing for the selection of superior individuals even before phenotypic data is available [1, 2]. Unlike traditional marker-assisted selection [3], which focuses on a few quantitative trait loci (QTLs), GS captures the cumulative effects of all available markers across the genome, leading to more accurate and efficient selection of elite genotypes [4–6]. As plant breeding programs increasingly incorporate GS, further research has been conducted to optimize the methods used for genomic prediction. Genomic prediction, just like other applications of statistical modeling, is characterized by two dominant modeling cultures: the data model culture and the algorithmic model culture [7]. These cultures reflect distinct approaches to handling genetic data and extracting predictive signals.

### The data model culture: structured statistical modeling [7]

The data model culture, primarily associated with classical statistical approaches, assumes an explicit, often linear relationship between genetic markers and phenotypic traits. Classical quantitative genetics often adopts the data modeling culture, using structured models that are motivated by genetic theory to estimate additive and dominance effects for genomic selection. Parametric models, such as genomic best linear unbiased prediction (GBLUP), are widely used [4, 5, 8–11]. GBLUP utilizes the genomic relationship matrix (G) and assumes that marker effects are normally distributed to calculate genomic breeding values, thereby providing a robust and interpretable framework for genomic selection. Several extensions of the data model culture have been developed to improve prediction accuracy, accommodate variable genetic architectures, integrate different data sources, and enhance computational efficiency. These include:

- GBLUP with additive, dominance, and/or epistasis: Extends the standard GBLUP model by incorporating a dominance relationship matrix (D) to capture dominance deviations and epistatic effects using Hadamard products of genomic matrices. The model can be flexibly applied to extend only dominance effects, only epistasis, or a combination of dominance and epistasis, providing a more comprehensive approach to genomic prediction [12–24].
- Ridge Regression Best Linear Unbiased Prediction (rrBLUP): A linear mixed model equivalent to GBLUP, in which genetic effects are estimated in marker space using ridge regression. It is widely used in genomic prediction because it handles multicollinearity among markers, with computational efficiency depending on the relative number of markers and individuals; rrBLUP is generally more computationally efficient when the number of individuals exceeds the number of markers [18, 25, 26].
- Bayesian models (e.g., BayesA, BayesB, BayesCπ): Introduce marker-specific shrinkage to allow for variable effect sizes and sparsity [27, 28].

While these methods provide structured and interpretable frameworks for genomic prediction, they are limited in their ability to capture complex, non-linear genetic interactions such as dominance and epistasis. This limitation arises from their reliance on predefined genetic structures, in which additive and dominance effects are typically modeled as separate components with limited flexibility to represent more complex dependencies, as well as from their linear assumptions. Moreover, the assumption that marker effects follow a normal distribution may not always hold, potentially leading to the underestimation of non-additive effects that contribute to phenotypic variation.

### The algorithmic model culture: machine learning and deep learning [7]

In contrast to the data model culture, the algorithmic model culture, primarily driven by machine learning (ML) and deep learning (DL), focuses on non-parametric, data-driven approaches that learn complex genetic patterns directly from data.

Examples of ML methods applied to genomic prediction include:

- Random Forests (RF): An ensemble learning method that captures non-linear interactions through decision trees. There have been various applications of random forest in plant breeding, with varying levels of success [26, 29–32].
- Support Vector Machines (SVMs): A kernel-based approach that maps genomic data into higher-dimensional spaces to capture complex relationships [33, 34])
- Boosting Machines: Gradient Boosting Machines (GBM) and their variants, such as Light gradient boosting machines (LightGBM) and Extreme gradient boosting machine (XGBoost), are widely used in plant breeding due to their ability to handle complex datasets and improve prediction accuracy. These methods sequentially enhance weak learners to create strong predictive models, making them well-suited for genomic selection and trait prediction [35–38].

Deep learning, particularly neural networks, has also gained significant traction due to its ability to model high-dimensional, non-linear interactions. Examples of deep learning methods applied in the genomic prediction of plants for breeding purposes include:

- Deep Neural networks: Learn hierarchical feature representations from genomic data without prior assumptions about genetic architecture [39–44].
- Convolutional Neural Networks (CNNs): Originally developed for analyzing grid-like data such as images, CNNs have been adapted for genomic prediction to exploit the sequential organization of genetic markers along chromosomes. In this context, “spatial patterns” refer to local dependencies among adjacent markers—such as linkage disequilibrium blocks or haplotype segments—rather than physical spatial positions. By applying convolutional filters across marker sequences, CNNs can automatically extract these locally correlated genomic features, thereby improving the prediction of complex traits in structured genomic data [45–48].

Despite the different levels of success observed in their application in Plant Breeding, ML, and DL models often require large datasets for effective training and suffer from limited interpretability. Furthermore, their reliance on black-box optimization makes it challenging to incorporate prior knowledge from quantitative genetic theory.

### A hybrid genomic modeling culture

Despite the successes of data and algorithmic model cultures in plant breeding, both cultures have limitations that restrict their ability to fully leverage genomic information and its applications in plant breeding. One such limitation is that the genomic data model’s variation attempts to incorporate different sources of variation using multiple kernels. Depending on the chosen model, genomic data models can capture additive, dominance, and epistatic effects, either individually or in combination, by using specific genomic relationship matrices or covariance structures as input. In such modeling, there is an assumption of independence between matrices, although this is a statistical assumption that may not fully capture the underlying genetic relationships. For genomic algorithmic models, interactions among loci are implicitly captured from the marker data without the need to construct separate relationship matrices or kernels. However, the process by which these models identify, represent, and use such interactions is not fully understood, leading to limited interpretability despite sometimes high predictive accuracy.

Here, we introduce a hybrid genomic modeling culture that integrates both data model and algorithmic model cultures (see *Materials and Methods*), aiming to harness their complementary strengths in addressing the assumption of independence among multiple kernels in traditional genomic models and in enhancing the interpretability of machine learning results. This culture combines structured statistical modeling with the flexibility of machine learning. Central to this approach is the 2NP matrix, a novel representation that concatenates the additive-centered matrix (Z) and the dominance-deviation matrix (W) to explicitly capture additive and dominance effects, respectively. The hybrid genomic modeling culture, which integrates 2NP with machine learning algorithms, provides a powerful means of modeling both additive and non-additive genetic effects, including their interactions. The concatenation of both matrices within the 2NP structure highlights the dependencies between additive and dominance components, while the machine learning model identifies and utilizes these interactions. This unified approach holds significant promise for enhancing selection accuracy and understanding complex genetic architectures in breeding programs. The machine learning method adopted in this study was LightGBM. Boosting approaches have consistently demonstrated strong performance in genomic prediction, often matching or outperforming alternative methods such as random forests, support vector machines, and deep learning models, particularly in structured datasets with moderate sample sizes [49–51]. While other machine learning methods could also be considered, the focus of this study was to evaluate the integration of the 2NP matrix within a high-performing non-linear method.

### Efficiency of selection

In a breeding program, selection represents the final component of the process in which genotypes with desirable traits are identified and retained from a population. In the context of GS, the efficiency of selection becomes a critical focus, as various models are employed to enhance the accuracy of predicting genetic merit [8]. Traditionally, GS is framed as the task of predicting an individual’s genomic estimated breeding value (GEBV) for a target trait [1, 2, 52]. While prediction accuracy is commonly used to evaluate model performance, breeders are often more concerned with practical outcomes, specifically, how well a model ranks and identifies the top-performing genotypes. Therefore, in this study, beyond assessing model performance through prediction accuracy, we also address a key breeding question: How efficiently does the model enable the selection of the best performers? To answer this, we evaluate the efficiency of selection, defined as the model’s ability to correctly identify individuals with the highest true performance. By bridging the gap between structured statistical approaches and machine learning methods, we aim to establish a hybrid culture (explained above) that improves selection efficiency while preserving interpretability of genetic effects.

## Materials and methods

### 2NP matrix theory: bridging the gap in the application of machine learning methods in genomic prediction

There are two main standard genomic best linear unbiased prediction (GBLUP) models: GBLUP model with additive genetic effects only (GBLUP_ADD), that assumes that the phenotypic value $\:(y$) is modeled as:

  

$$y=Xb+{Z_A}a+e$$

and GBLUP model incorporating both additive and dominance genetic effects (GBLUP_ADDOM), that assumes that the phenotypic value $\:(y$) is modeled as:

$$\:y=Xb+{Z}_{A}a+{Z}_{D}d+e$$

where $\:y$ is the vector of phenotypes$\:\:\:X$, $\:{Z}_{A}\:$ and $\:{Z}_{D}$are incidence matrices for $\:b$, $\:a,$ and $\:d$, respectively. $\:b$ is the solution vector of fixed effects (including the overall mean), $\:a\:\sim\:N\left(0,A{\sigma\:}_{a}^{2}\:\right)$ represents the additive genetic effects,$\:\:d\:\sim\:N\left(0,D{\sigma\:}_{d}^{2}\:\right)$represents the dominance genetic effects, $\:\:e\:\sim\:N\left(0,I{\sigma\:}_{e}^{2}\right)$ is the vector of residual effects, $\:A$ is the additive genomic relationship matrix, $\:D$is the dominance genomic relationship matrix, $\:I$ is the identity matrix, $\:{\sigma\:}_{A}^{2}$, $\:{\sigma\:}_{D}^{2}$, $\:{\sigma\:}_{e}^{2}$ are the additive genetic variance, dominance genetic variance, and residual variance, respectively.

### Genomic relationship matrices in standard GBLUP

The additive genomic relationship matrix ($\:A$) is estimated following [2]:

$$\:A\:=Z{Z}^{{\prime\:}}{\left({\sum\:}_{i=1}^{m}2{p}_{i}\left(1-2{p}_{i}\right)\right)}^{-1}$$

where $\:Z\:=\:M\:\--\:P\:$; is the centered genotype matrix, $\:M\:$ is the marker matrix coded as 0,1,2 for alternative alleles, $\:P\:=\:{{2p}_{i}^{}}_{}$is the matrix of allele frequencies, $\:{{p}_{i}^{}}_{}$is the allele frequency at locus *i*.

The dominance genomic relationship matrix *(*$\:D$*)* is estimated using [13]:

$$\:D\:=W{W}^{{\prime\:}}{\left({\sum\:}_{i=1}^{m}4{p}_{i}^{2}{q}_{i}^{2}\right)}^{-1}$$

where $\:W$ is a matrix of heterozygosity coefficients; the $\:i$-th column of $\:\:W$ is defined as $\:-{{2p}_{i}^{2}}_{}$for homozygous alleles and $\:{2p}_{i}{q}_{i}$ for heterozygotes, where $\:{p}_{i}$ and $\:{q}_{i\:}=\:1-{p}_{i}\:$ are allele frequencies at locus$\:\:i$, respectively [13].

### Limitations of standard GBLUP and the 2NP matrix approach with gradient boosting integration

**Fig. 1:**
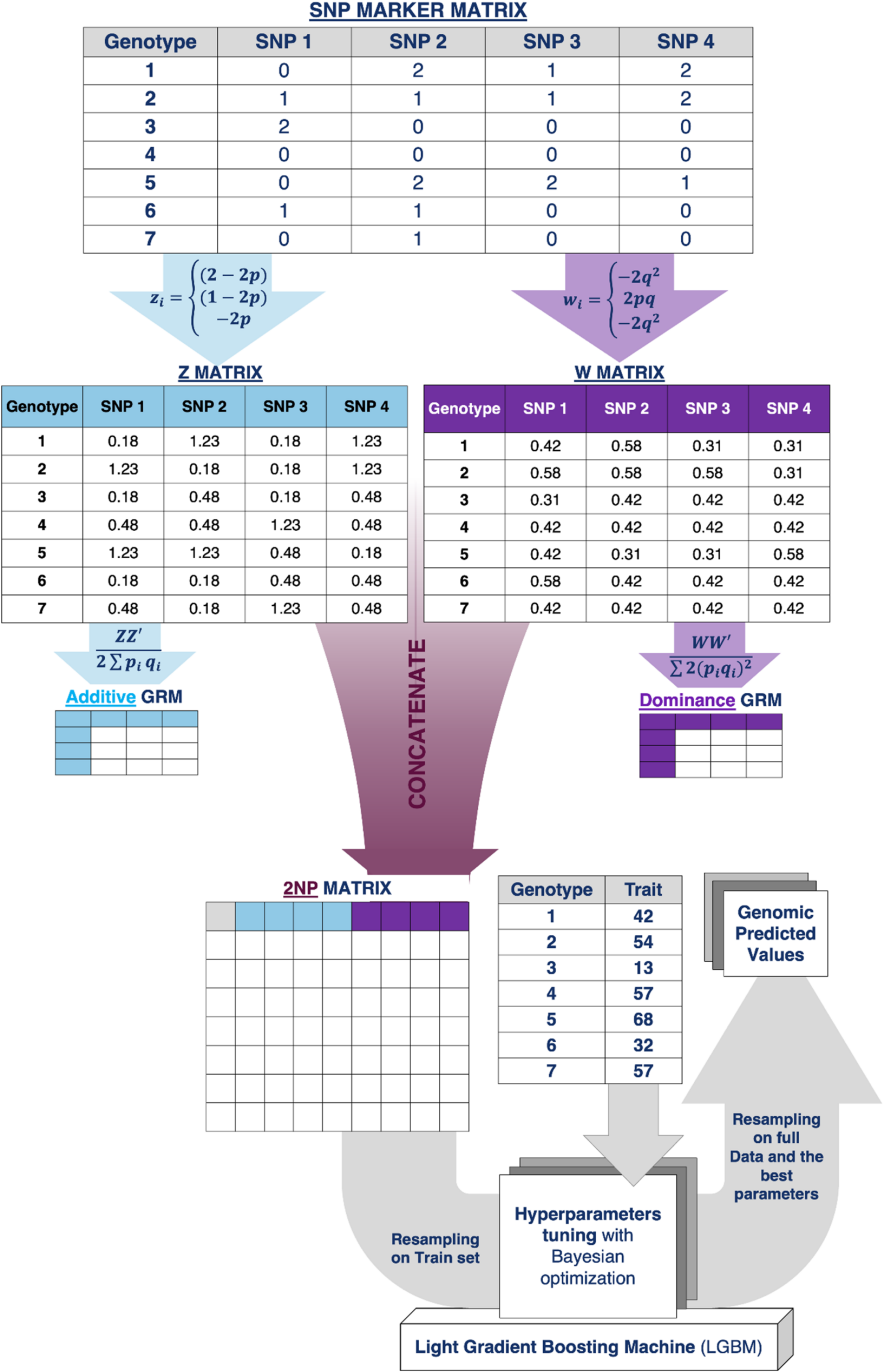
Visual workflow of the 2NPLGBM genomic prediction model. The process begins with a SNP marker matrix, where individuals are genotyped across multiple loci. The marker matrix is transformed into an additive matrix (Z) and a dominance matrix (W) based on allele frequency coding. These matrices are concatenated to form the 2NP matrix, which jointly represents additive and dominance effects. For comparison, in conventional two-kernel GBLUP models, additive and dominance genomic relationship matrices (GRMs) are typically derived from Z and W, respectively. The 2NP matrix, together with phenotypic trait data, is used as input for a Light Gradient Boosting Machine (LGBM). Model training involves resampling of the training set and hyperparameter tuning via Bayesian optimization, after which the optimized model is applied to generate predicted (estimated) values

The GBLUP model assumes that additive and dominance effects are independent, although this simplification may not fully capture their underlying relationships. The interactions play an important role in trait expression. To address this limitation, we propose the 2NP matrix, which concatenates the additive-centered matrix ($\:Z$) and the dominance deviation matrix ($\:W$) into a single combined kernel:

$$ \:G_{{2NP}} \: = \:\left[ {\:Z|W} \right] $$

where $\:Z\:$captures additive genetic effects and $\:W$ captures dominance deviations. This combined structure maintains the individual contributions of additive and dominance variance while enabling interactions to emerge naturally. While the 2NP matrix captures both additive and dominance effects, gradient boosting methods (e.g., LGBM and XGBoost) are leveraged to model complex interactions. The prediction model now takes the form:

$$\:y\:=\:f\left({G}_{2NP}\right)+\:e\:$$

where $\:f\left({G}_{2NP}\right)$ is a gradient-boosting machine that captures additive effects $\:{g}_{A}$​, dominance effects $\:{g}_{D}$​, additive-additive interactions $\:{g}_{AA}$, additive-dominance interactions $\:{g}_{AD}$​, and dominance-dominance interactions $\:{g}_{DD}$​. The gradient boosting model optimizes a non-linear transformation of $\:{G}_{2NP}$​, capturing higher-order interactions between loci that are typically ignored in standard GBLUP Fig. 1.

### Phenotypic and genotypic data

We used data from Genomes to Fields (G2F) 2024 Maize Genotype by Environment Prediction Competition [53]. We used data spanning 6 years, from 2018 to 2023, which consisted of 2,925 unique maize (*Zea mays L.*) hybrids evaluated in multiple environments across the United States, Canada, and Germany. The modified Randomized Complete Block Design (RCBD), mainly with two replications per environment, was used in the trials. Our analysis covers four traits: Plant Height (cm), Days to Anthesis (days), Days to Silking (days), and Grain Yield (Mgha^− 1^).

The genotypic data were described in [53]. For the G2F materials from 2014 to 2023, variant calls were performed using the Practical Haplotype Graph (PHG) [54]. Hybrid genotypes were generated by combining information from their parent lines using the CreateHybridGenotypes plugin available in TASSEL 5 [55], yielding 5,899 individuals. SNPs with a minor allele frequency (MAF) below 1% were then filtered out, resulting in 2,425 high-quality variant positions. We filtered for the years 2018, 2019, 2020, 2021, 2022, and 2023. Since the SNP markers were already filtered, there was no need for further filtering.

To identify and remove outliers, a linear model was fitted with the hybrids and replicates as fixed effects in each unique environment, defined by field location and year, as described by [56]. To reduce computational time, a two-step analysis was employed to calculate the best linear unbiased estimates (BLUEs) for each hybrid, as described in [56]. To generate the best linear unbiased estimates (BLUEs) for each hybrid in each environment, we adjusted the BLUEs from the first of the twosteps, with a linear mixed model considering hybrid as a fixed effect and field location (FL) as a random effect:

$$\:{y}_{if}=\mu\:\:+{H}_{i}+{FL}_{f}+{e}_{if}\:$$

where $\:{y}_{if}$ is the BLUE of the *i*-th hybrid calculated from (the first step); $\:\mu\:$ is the overall mean; $\:{H}_{i}$ is the fixed effect of the-ℎ hybrid; $\:{FL}_{f}$is the random effect of the *f*-th field location; $\:{e}_{if}$ is the residual term associated with the observation$\:{\:y}_{if}$ .

### Heritability estimation

Additive [2] and dominance [13] genomic relationship matrices were constructed using the AGHmatrix package [57] in R version 4.2.2 [58]. These matrices were used to model additive and dominance genetic effects in GBLUP. Variance components were estimated using the EMMREML package [59]. Specifically, the *emmremlMultiKernel* function was used to fit mixed models with multiple covariance structures (in our case, additive and dominance kernels), whereas the *emmreml* function is used for models with a single covariance structure.

The proportion of dominance variance was calculated relative to the total genotypic variance as:

- Proportion of dominance variance (genotypic scale):

$$\:PDV=\frac{{V}_{d}}{{V}_{g}}$$

and relative to the total phenotypic variance as:

- Proportion of dominance variance (phenotypic scale):

$$\:{d}^{2}=\frac{{V}_{d}}{{V}_{p}}$$

Narrow-sense heritability was calculated as:

- Narrow-sense heritability:

$$\:{h}^{2}=\frac{{V}_{a}}{{V}_{p}}$$

and broad-sense heritability was calculated as:

- Broad-sense heritability:

​

$$\:{H}^{2}=\frac{{V}_{a}+{V}_{d}}{{V}_{p}}$$

where$\:{\:V}_{g}=\:{V}_{a}+{V}_{d}$ ​represents the total genotypic variance and $\:{V}_{p}=\:{V}_{a}+{V}_{d}+\:{V}_{e}$ denotes the total phenotypic variance. And $\:{V}_{a}$, $\:{V}_{d}$, and $\:{V}_{e}$ denoting additive, dominance and residual variance components, respectively.

### Genomic prediction models

Classical models: Two GBLUP models were chosen for this study. These are the GBLUP model, which utilizes a single genomic relationship matrix for additive effects [2], and the GBLUP model, which employs a genetic relationship matrix for both additive and dominance effects, using separate kernels for each [13]. Both have already been described above.

Machine Learning Models: The 2NP genomic prediction model was fitted using a Gradient Boosting Machine (GBM) implemented in the LightGBM framework [60] within in Python 3.10 [61]. The model was trained using a structured workflow that combined automated Bayesian hyperparameter optimization with the LightGBM machine learning algorithm. Hyperparameter tuning was performed using the *BayesSearchCV* function from the Scikit-Optimize library [62] which applies sequential model-based optimization to efficiently explore the hyperparameter space while reducing the risk of overfitting. For each cross-validation (CV) scheme (LOYO, CV0, CV00, 5-fold, and Rolling Window. See description of CV schemes below), optimization was conducted exclusively within the training set to prevent information leakage. A total of 20 Bayesian optimization iterations were performed using five-fold internal CV and mean squared error (MSE) as the scoring metric. The optimal hyperparameter configuration identified from the training data was then used to fit the final model, and predictive performance was evaluated on the corresponding test set. Details of the hyperparameter search space are provided in Supplementary Material 1 (Sheet 1). The above process was also followed for the LightGBM model with standard SNP marker matrix.

Model interpretability was achieved using SHapley Additive exPlanations (SHAP) [63], which quantified the contribution of each genomic feature to prediction outcomes.

The results of 2NPLGBM were compared with those of GBLUP. Two GBLUP models were chosen for this study. The GBLUP model (GBLUP_ADD), which uses a single genomic relationship matrix for additive effects [2], and the GBLUP model (GBLUP_ADDOM), which uses a genetic relationship matrix for both the additive and dominance effects using separate kernels for each [13].

### Validation schemes

To evaluate the model’s performance and show realistic scenarios, we implemented five distinct validation schemes: two designed to simulate unseen years, Leave-One-Year-Out (LOYO) and Rolling Window (RW), and three designed to capture genetic relationships, Five-Fold, Tester CV0, and Tester CV00. For the schemes based on genetic relationships, we conducted 10 repetitions of five-fold cross-validation. In each repetition, the phenotypic data were partitioned into five subsets; each subset served as the validation set once, while the remaining four were used for training [64, 65].

#### LOYO

In the LOYO scheme, data from all but one year were used for training, while the left-out year served as the test set. We utilized data from all six years (2018–2023), resulting in a total of six validations, one for each year.

#### RW

In the Rolling Window scheme, a fixed window of three consecutive years was used as the training set, and the following year was used as the test set. This window was then shifted forward by one year at a time, and the procedure was repeated until the last year (2023).

#### Five-fold

For the Five-Fold scheme, hybrid genotypes were randomly divided into five equal-sized folds. In each round of cross-validation, four folds were used for training, and the remaining fold was used for testing. This process was repeated 10 times to ensure robustness.

#### Tester CV0

The Tester CV0 scheme focused on predicting hybrids with known testers in a new year. As with CV0, models were trained using trials from 2018 to 2022 and tested on trials from 2023. In each fold, 20% of the testers evaluated in 2023 were sampled to create the test set, and these 20% testers were retained and included in the training set.

#### Tester CV00

The Tester CV00 scheme aimed to predict the performance of hybrids involving unknown testers in a new year. Models were trained on trials from 2018 to 2022 and tested on trials from 2023. In each fold, 20% of the testers evaluated in 2023 were sampled to form the test set, and these 20% randomly chosen testers were excluded from the training data across years in the training set.

### Model performance metrics

Accurate prediction of genetic merit is a cornerstone of genomic selection (GS), enabling breeders to make informed decisions about which Genotype to advance. While several statistical and machine learning models have been developed to enhance predictive power, their utility ultimately depends on how well they capture the relationship between genotypic and phenotypic variation.

In this study, we evaluated the model’s performance using two metrics: Pearson’s correlation coefficient and Selection Efficiency. Pearson’s correlation measures the linear association between observed phenotypes and predicted phenotypes, providing a standard indicator of prediction accuracy. However, in practical breeding applications, accurate ranking of individuals is often more critical than raw prediction accuracy. To address this, we also employed selection efficiency [65–67], which evaluates how well a model identifies top-performing genotypes. We measured the selection efficiency considering the top percentage (5%, 10%, 20%, 30%) of the hybrid genotype. It is calculated as:

$$ \:{\mathrm{Selection}}\:{\mathrm{Efficiency}}\: = \:\frac{{I\: - \:C}}{{N\: - \:C}} $$

where:

- N is the total number of individuals evaluated,
- I is the number of individuals common to both observed and predicted top % sets, and.
- C is the expected number of overlaps by random chance (i.e., the expected number of individuals selected by chance).

## Results

### Population structure of the hybrids from 2018 to 2023

After data cleaning, 2,425 SNP markers were retained for downstream analyses. Between 2018 and 2023, a total of 2,925 unique hybrids were evaluated, with the highest number recorded in 2021 (1,180) and the lowest in 2023 (546) (see Table 1). During this period, 1,495 genotypes (as female parents) and 38 genotypes (as male parents) were used. The number of genotypes varied across years, peaking in 2019 (603 genotypes) and gradually declining thereafter. In contrast, the number of testers remained relatively stable from 2020 to 2023, at 18 per year, following a peak of 27 testers in 2018 (see Table 1). Among the male parents used each year, there were major testers: 2 in 2018 and 2019 (LH195, PHT6), 3 in 2020 and 2021 (PHZ51, PHK76, PHP02), and 1 in 2022 and 2023 (LH244).

**Fig. 2:**
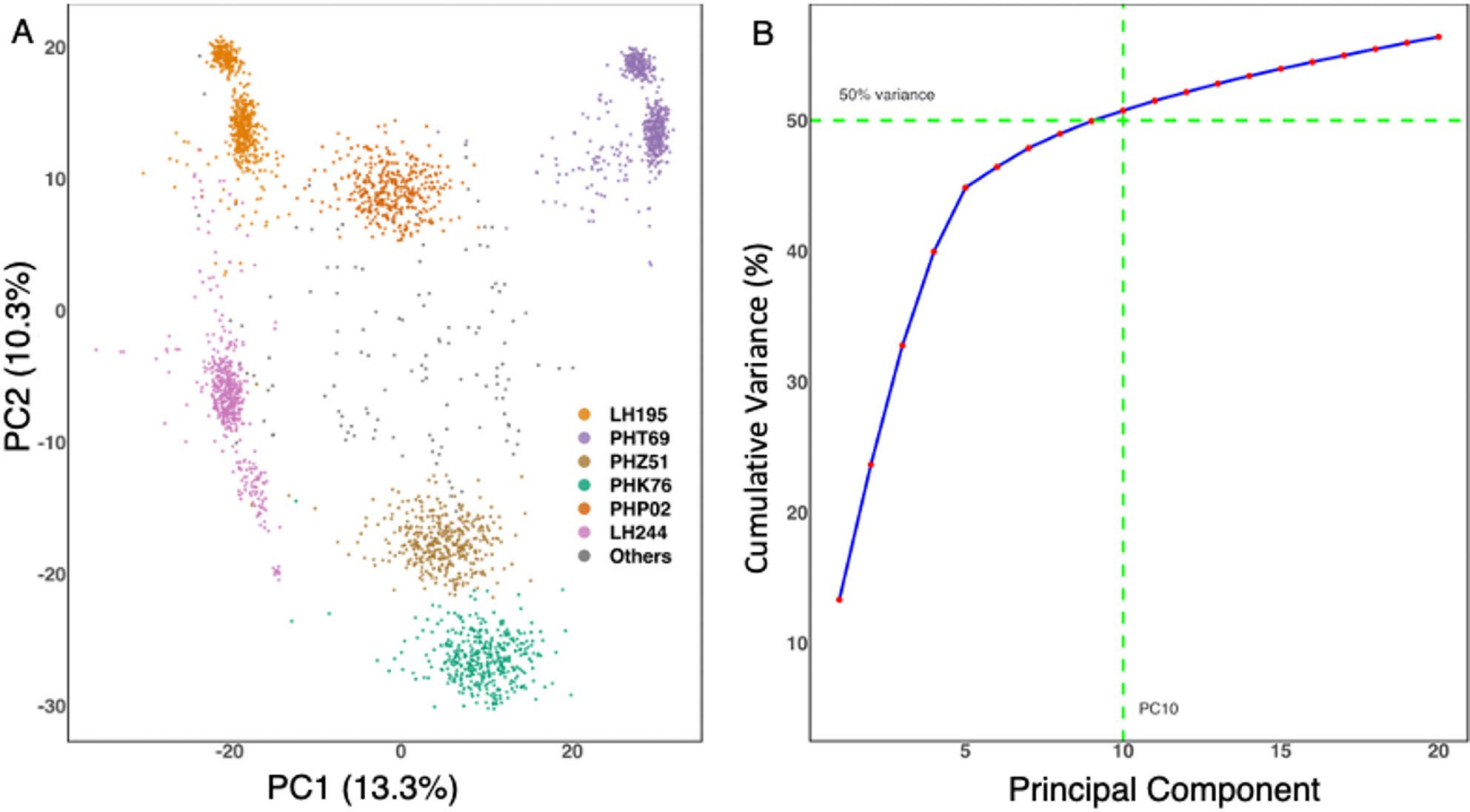
Principal Component Analysis (PCA) of SNP data in the hybrid maize population. **A** Genetic structure showing clusters primarily associated with the tester used in hybrid development. **B** Cumulative variance explained by the first 20 principal components, with the first 10 PCs accounting for just over 50% of the total genetic variance. The first two PCs explain 23.6% of the variation, revealing seven distinct genetic groups

A Principal Component Analysis (PCA) of the SNP data revealed clear genetic structure within the test-cross hybrid population, with clusters primarily reflecting the tester used in hybrid development (Fig. 2a). The first 10 principal components together explained just over 50% of the total genetic variance (Fig. 2b), indicating a substantial underlying structure among the hybrids across years. Based on the PCA, seven distinct genetic groups were identified, with the first two principal components accounting for 23.6% of the variation.

**Table 1:** Summary of the hybrid maize population evaluated across six years (2018–2023). For each year, the number of hybrids, parental lines, and testers used is shown

Year	Hybrid parent 1	Hybrid parent 2	Number of hybrids	Major tester
2018	578	27	1039	LH195, PHT69
2019	601	17	1158	LH195, PHT69
2020	403	18	1175	PHZ51, PHK76, PHP02
2021	408	18	1180	PHZ51, PHK76, PHP02
2022	525	18	549	LH244
2023	522	18	546	LH244

### Comparison of model performance across four traits and two different CV scenarios, simulating unseen years

The scenarios to be simulated were done in two different ways: Leave One Year Out (LOYO) and Rolling Window (RW).

#### LOYO

**Fig. 3:**
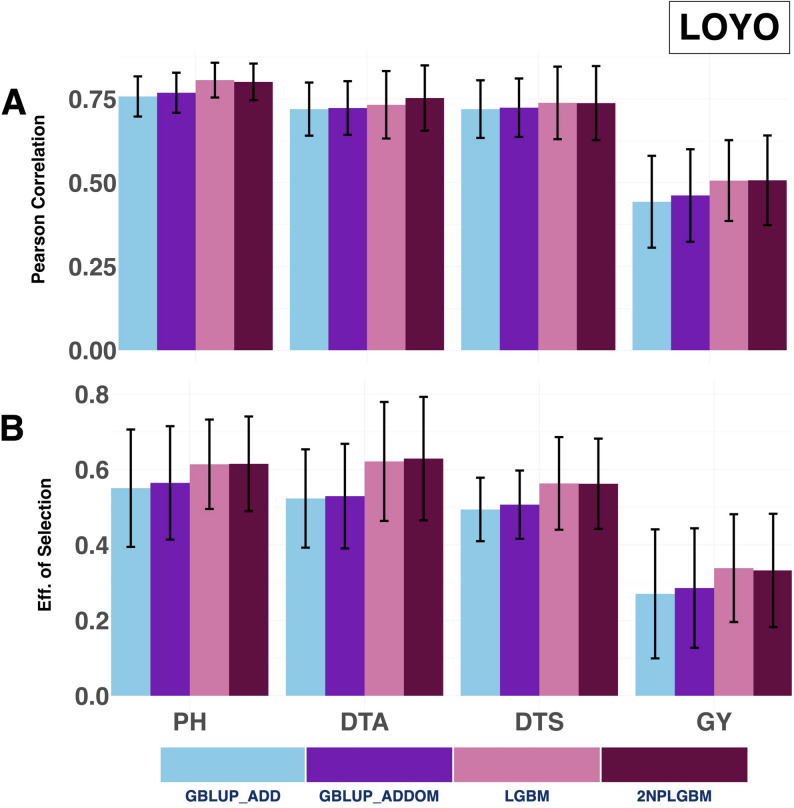
Prediction ability and selection efficiency for four agronomic traits, Grain Yield, Plant Height, Days to Anthesis, and Days to Silking, were evaluated under the LOYO validation scheme. The performance of four genomic prediction models is compared: the GBLUP additive model (GBLUP_ADD), the GBLUP additive + dominance model (GBLUP_ADDOM), the standard LightGBM model (LGBM), and the 2NP matrix-based LightGBM model (2NPLGBM). Predictive ability is expressed as the mean Pearson correlation between observed and predicted phenotypes, with error bars representing the standard error across years. Selection efficiency, measured by the accuracy in identifying the top 20% performing hybrids, is similarly shown with standard error bars

When the objective was to predict hybrid performance in a new year (LOYO), both machine learning models (LGBM and 2NPLGBM) outperformed both GBLUP models tested in this study across all four traits. The average prediction accuracies achieved by the 2NPLGBM model were 0.507 for Grain Yield, 0.737 for Days to Silking, 0.754 for Days to Anthesis, and 0.801 for Plant Height (Fig. 3 & Supplementary Material 1 (Sheet 2). The LGBM model showed comparable performance, with slightly higher accuracy for Plant Height (0.806) and similar performance for Grain Yield (0.506) and Days to Silking (0.738), but lower accuracy for Days to Anthesis (0.733). A similar trend was observed for selection efficiency, with the 2NPLGBM model also demonstrating superior performance, yielding average values of 0.332 for Grain Yield, 0.562 for Days to Silking, 0.629 for Days to Anthesis, and 0.615 for Plant Height while LGBM achieved comparable values of 0.338, 0.563, 0.621, and 0.614, respectively. Furthermore, both machine learning models achieved an increase in prediction accuracy and selection efficiency compared to the GBLUP_ADD across traits. The 2NPLGBM model achieved a 2% to 14% increase in prediction accuracy across traits, while LGBM showed similar gains (approximately 2% to 14%). In terms of selection efficiency, improvements ranged from 11% to 20% for 2NPLGBM, with LGBM showing comparable gains for some traits.

#### RW

**Fig. 4:**
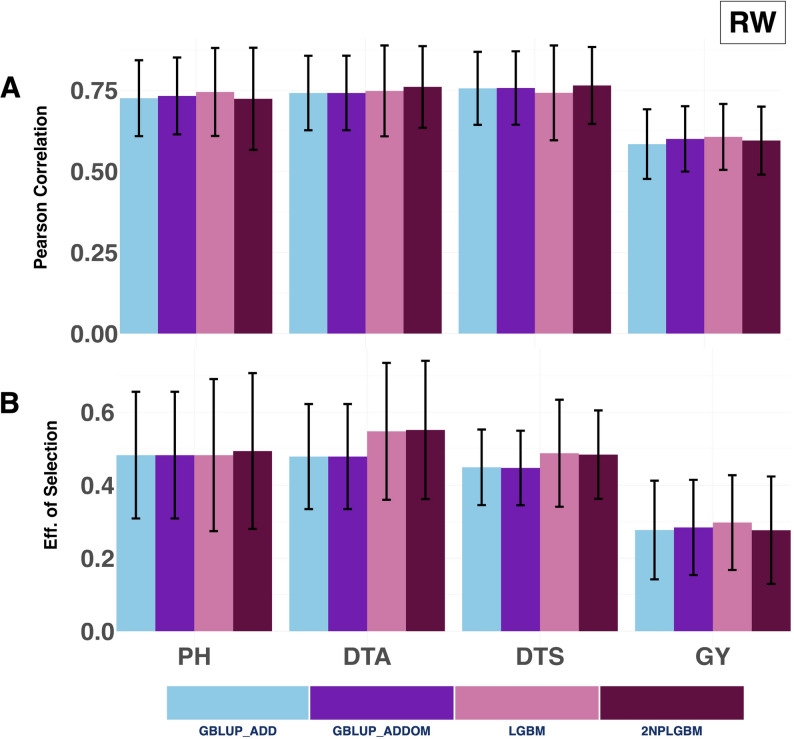
Prediction ability and selection efficiency for four agronomic traits, Grain Yield, Plant Height, Days to Anthesis, and Days to Silking, were evaluated under the RW validation scheme. The performance of four genomic prediction models is compared: the GBLUP additive model (GBLUP_ADD), the GBLUP additive + dominance model (GBLUP_ADDOM), the standard LightGBM model (LGBM), and the 2NP matrix-based LightGBM model (2NPLGBM). Predictive ability is expressed as the mean Pearson correlation between observed and predicted phenotypes, with error bars representing the standard error across years. Selection efficiency, measured as the accuracy of identifying the top 20% performing hybrids, is similarly shown with standard error bars

The results from the rolling window (RW) validation scheme differed from those observed under the LOYO validation scheme. For average prediction accuracy, the GBLUP_ADDOM model showed strong performance for Grain Yield (0.600), while LGBM achieved the highest accuracy for both Grain Yield (0.606) and Plant Height (0.744), outperforming 2NPLGBM and GBLUP_ADD. In contrast, for Days to Silking and Days to Anthesis, the 2NPLGBM model showed superior performance, with average accuracies of 0.764 and 0.760, respectively (Fig. 4 & Supplementary Material 1 (Sheet 3)).

A similar trend was observed for selection efficiency. The 2NPLGBM model demonstrated the largest improvement for Days to Anthesis, achieving an increase of approximately 15% relative to GBLUP_ADD. It also showed competitive performance for Days to Silking and Plant Height, with average selection efficiencies of 0.484 and 0.493, respectively, comparable to or slightly higher than those of LGBM. In contrast, for Grain Yield, LGBM achieved the highest selection efficiency (0.298), while 2NPLGBM showed no improvement relative to GBLUP_ADD (Fig. 4 & Supplementary Material 1 (Sheet 3)).

### Comparison of model performance across four traits and three different CV scenarios, simulating different genetic relationships

#### Five-Fold

**Fig. 5:**
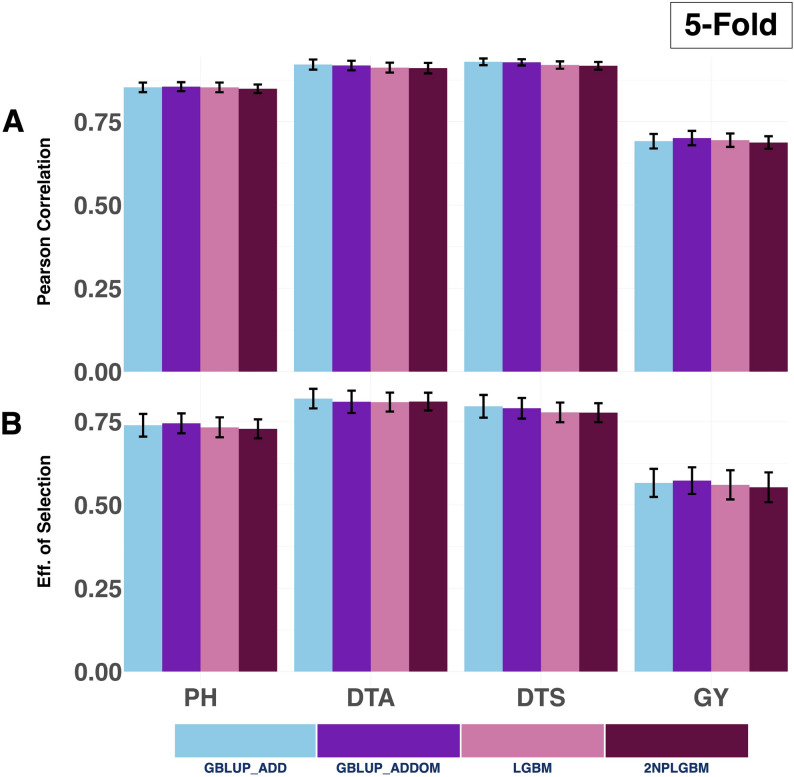
Prediction ability and selection efficiency for four agronomic traits, Grain Yield, Plant Height, Days to Anthesis, and Days to Silking, were evaluated under the 5-Fold cross-validation scheme. The performance of four genomic prediction models is compared: the GBLUP additive model (GBLUP_ADD), the GBLUP additive + dominance model (GBLUP_ADDOM), the standard LightGBM model (LGBM), and the 2NP matrix-based LightGBM model (2NPLGBM). Predictive ability is expressed as the mean Pearson correlation between observed and predicted phenotypes, with error bars representing the standard error across replicates. Selection efficiency, measured as the accuracy of identifying the top 20% performing hybrids, is similarly shown with standard error bars

Under the Five-fold cross-validation scheme, the 2NPLGBM model did not outperform either of the GBLUP models (GBLUP_ADD and GBLUP_ADDOM) and its machine learning counterpart (LGBM) when evaluated across all four traits tested. Among the models tested, GBLUP_ADDOM consistently achieved the highest prediction accuracy and selection efficiency for Plant Height and Grain Yield, while GBLUP_ADD achieved better performance for Days to Anthesis and Days to Silking. This result directly indicates that the GBLUP_ADD model, which uses only additive genetic relationships for prediction, favors traits with high heritability and low dominance, albeit in this cross-validation scheme (Fig. 5; Table 1 and Supplementary Material 1 (Sheet 4)). Both machine learning models (LGBM and 2NPLGBM) showed slightly lower prediction accuracies and selection efficiencies compared to the GBLUP-based approaches. LGBM consistently performed marginally better than 2NPLGBM across all traits, although the differences between the two were small, indicating broadly comparable performance.

#### Tester CV0

**Fig. 6:**
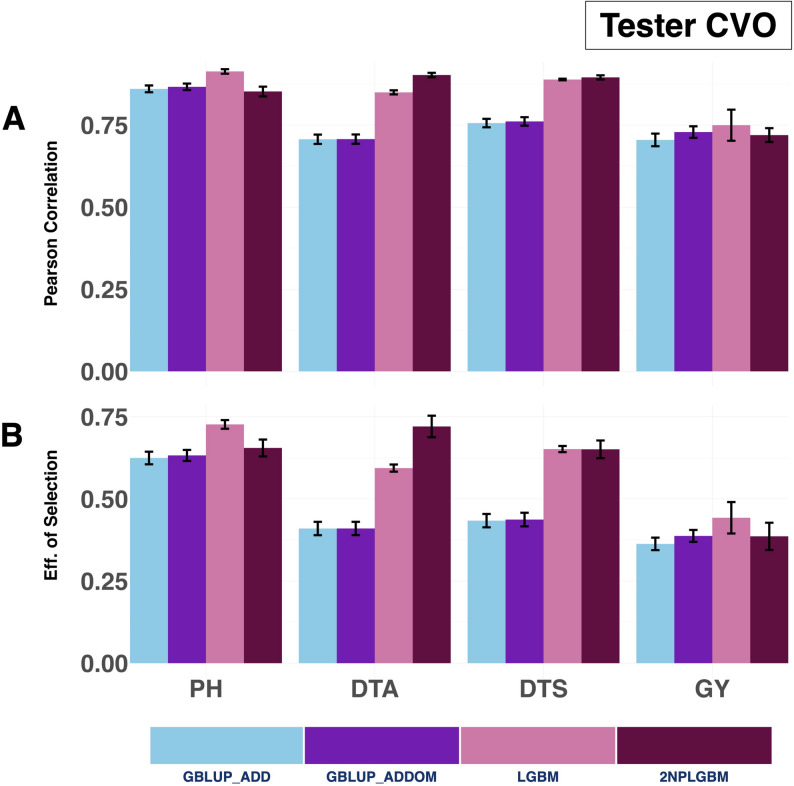
Prediction ability and selection efficiency for four agronomic traits, Grain Yield, Plant Height, Days to Anthesis, and Days to Silking, evaluated under the Tester CV0 cross-validation scheme. The performance of four genomic prediction models is compared: the GBLUP additive model (GBLUP_ADD), the GBLUP additive + dominance model (GBLUP_ADDOM), the standard LightGBM model (LGBM), and the 2NP matrix-based LightGBM model (2NPLGBM). Predictive ability is expressed as the mean Pearson correlation between observed and predicted phenotypes, with error bars representing the standard error across replicates. Selection efficiency, measured as the accuracy of identifying the top 20% performing hybrids, is similarly shown with standard error bars

Across the four traits evaluated, the 2NPLGBM model consistently outperformed the GBLUP models for flowering traits. For Days to Anthesis, 2NPLGBM achieved a Pearson correlation of 0.902, representing a 27.7% improvement over GBLUP_ADD (0.710). Similarly, for Days to Silking, 2NPLGBM had a Pearson correlation of 0.895, which is an 18.4% increase over GBLUP_ADD (0.755). In both cases, 2NPLGBM also outperformed the standard LGBM model, which achieved 0.850 for Days to Anthesis and 0.889 for Days to Silking. In contrast, for Plant Height, LGBM achieved the highest prediction accuracy (0.913), clearly outperforming both GBLUP_ADD (0.860) and GBLUP_ADDOM (0.867), while 2NPLGBM (0.852) showed a slight decline (-0.95%) relative to GBLUP_ADD. A similar pattern was observed for Grain Yield, where LGBM again performed best (0.749), followed by GBLUP_ADDOM (0.729), while 2NPLGBM (0.721) showed a modest improvement (2.08%) over GBLUP_ADD (0.705) (Fig. 6 & Supplementary Material 1 (Sheet 5)).

The 2NPLGBM model also demonstrated strong gains in selection efficiency, particularly for flowering traits. It achieved a 75.8% improvement for Days to Anthesis and a 50.0% increase for Days to Silking compared to GBLUP_ADD, outperforming both GBLUP models and standard LGBM. For Plant Height, however, LGBM showed the highest selection efficiency (0.726), exceeding both 2NPLGBM (0.655) and GBLUP-based models. For Grain Yield, LGBM again performed best (0.442), while 2NPLGBM (0.386) still improved upon GBLUP_ADD (0.363), and GBLUP_ADDOM showed a comparable performance (0.387) (Fig. 6 & Supplementary Material 1 (Sheet 5)).

#### Tester CV00

**Fig. 7:**
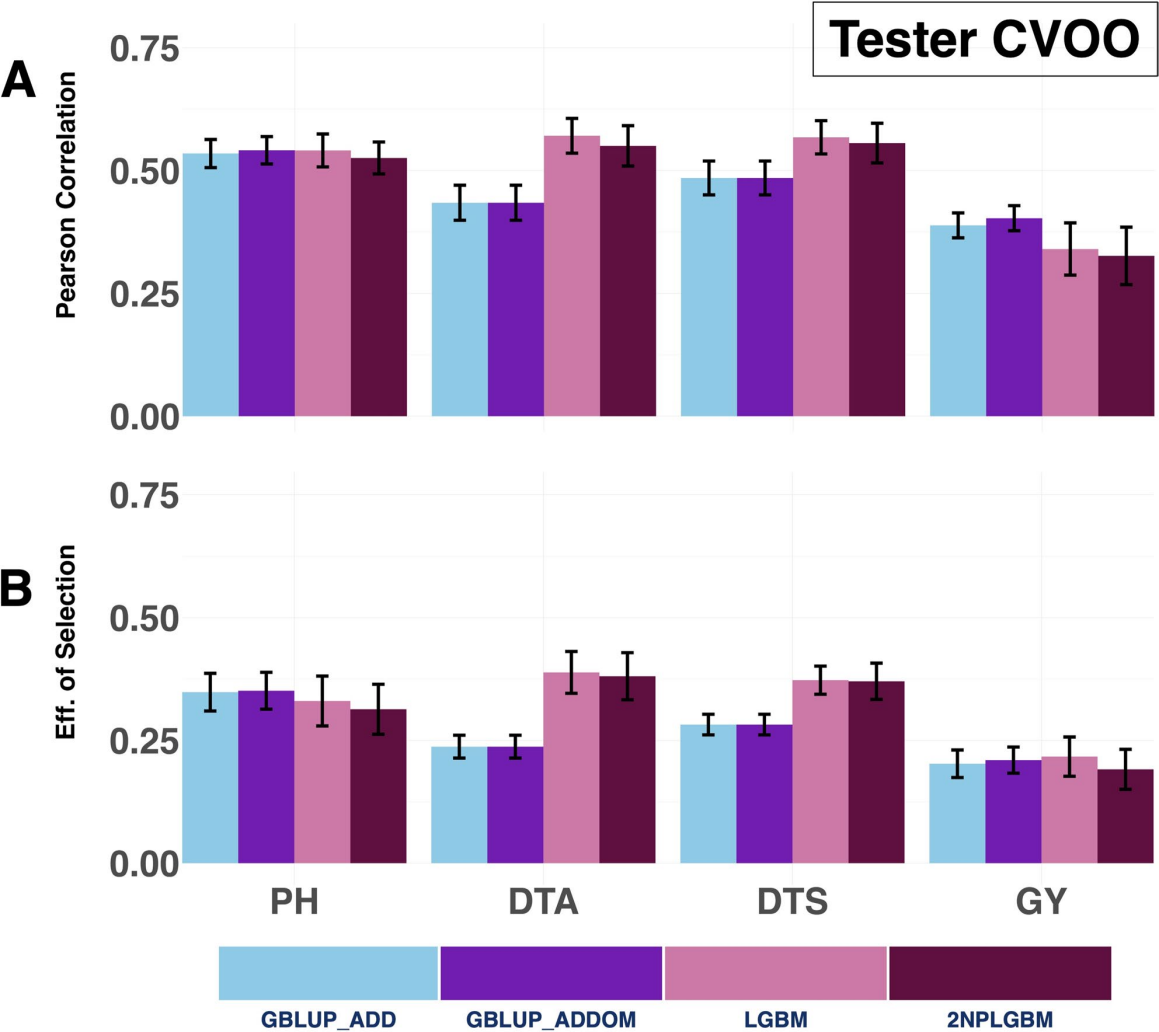
Prediction ability and selection efficiency for four agronomic traits, Grain Yield, Plant Height, Days to Anthesis, and Days to Silking, evaluated under the Tester CV00 cross-validation scheme. The performance of four genomic prediction models is compared: the GBLUP additive model (GBLUP_ADD), the GBLUP additive + dominance model (GBLUP_ADDOM), the standard LightGBM model (LGBM), and the 2NP matrix-based LightGBM model (2NPLGBM). Predictive ability is expressed as the mean Pearson correlation between observed and predicted phenotypes, with error bars representing the standard error across replicates. Selection efficiency, measured by the accuracy in identifying the top 20% performing hybrids, is similarly shown with standard error bars

Under this challenging scenario, all models showed lower predictive accuracy and selection efficiency across all traits. For Days to Anthesis and Days to Silking, both machine learning models outperformed the GBLUP approaches. The 2NPLGBM model achieved correlations of 0.55 and 0.56, respectively, substantially exceeding GBLUP_ADD and GBLUP_ADDOM (0.43–0.49). However, LGBM showed slightly higher predictive accuracy than 2NPLGBM for these traits (0.57 for Days to Anthesis and 0.57 for Days to Silking), although the differences were modest. In contrast, for Plant Height and Grain Yield, GBLUP_ADDOM performed best, with Pearson correlations of 0.54 and 0.40, respectively, outperforming both machine learning models. The performance of 2NPLGBM (0.53 for Plant Height and 0.33 for Grain Yield) and LGBM (0.54 and 0.34, respectively) was comparatively lower, particularly for Grain Yield (Fig. 7 & Supplementary Material 1 (Sheet 6)).

A similar pattern was observed for selection efficiency. Both 2NPLGBM and LGBM showed clear improvements over GBLUP for Days to Anthesis and Days to Silking, with gains exceeding 30–60% relative to GBLUP_ADD. In contrast, GBLUP_ADDOM achieved the highest selection efficiency for Plant Height and Grain Yield, while both machine learning models showed slightly reduced performance for these traits (Fig. 7 & Supplementary Material 1 (Sheet 6)).

### Trait heritability and the role of dominance effects

The partitioning of genetic variance revealed important differences in the relative contribution of additive and dominance effects across traits. Narrow-sense heritability (h^2^) was highest for Days to Silking (0.900) and Days to Anthesis (0.895), followed by Plant Height (0.780), and lowest for Grain Yield (0.476). The corresponding broad-sense heritability (H^2^) values were moderately higher, indicating low non-additive genetic components. Notably, the proportion of dominance variance accounted for 8.2% of total genetic variance in Plant Height, 11.1% in Days to Anthesis, 9.7% in Days to Silking, and 17.3% in Yield, which also exhibited the most dominance variance in relation to the broad sense heritability ($\:{d}^{2}$=0.121) (see Table 2).

**Table 2:** Narrow-sense heritability (h²), broad-sense heritability (H²), dominance variance proportion (d² = dominance variance / total phenotypic variance), and proportion of dominance variance (PDV = dominance variance / total genetic variance) for the four agronomic traits evaluated in the hybrid maize population

Trait	$\:{h}^{2}$	$\:{H}^{2}$	$\:{d}^{2}$	PDV
Plant Height (cm)	0.780	0.862	0.072	0.082
Days to Anthesis (days)	0.895	0.923	0.098	0.111
Days to Silking (days)	0.900	0.934	0.087	0.097
Grain Yield (Mgha^− 1^)	0.476	0.656	0.121	0.173

These patterns help explain the performance of different prediction models. The superior performance of 2NPLGBM and LGBM in predicting flowering traits (Days to Anthesis and Days to Silking) is consistent with their high heritability and meaningful, yet moderate, dominance contributions. In contrast, Grain Yield, while having lower additive heritability, exhibited the highest proportion of dominance variance, suggesting that incorporating non-additive effects is especially relevant for this trait. Nevertheless, 2NPLGBM did not outperform GBLUP models for grain yield prediction, indicating that dominance alone may not be sufficient and that other sources of complexity, such as environmental interactions, could further influence prediction accuracy.

Together, these results suggest that the advantages of using 2NPLGBM for genomic prediction may be trait-specific and appear more pronounced for traits with substantial dominance variance and relatively high heritability, as observed for flowering time traits in this study. However, further evaluation across additional datasets and traits is required to assess the generality of this observerd pattern.

### Variable importance

**Fig. 8:**
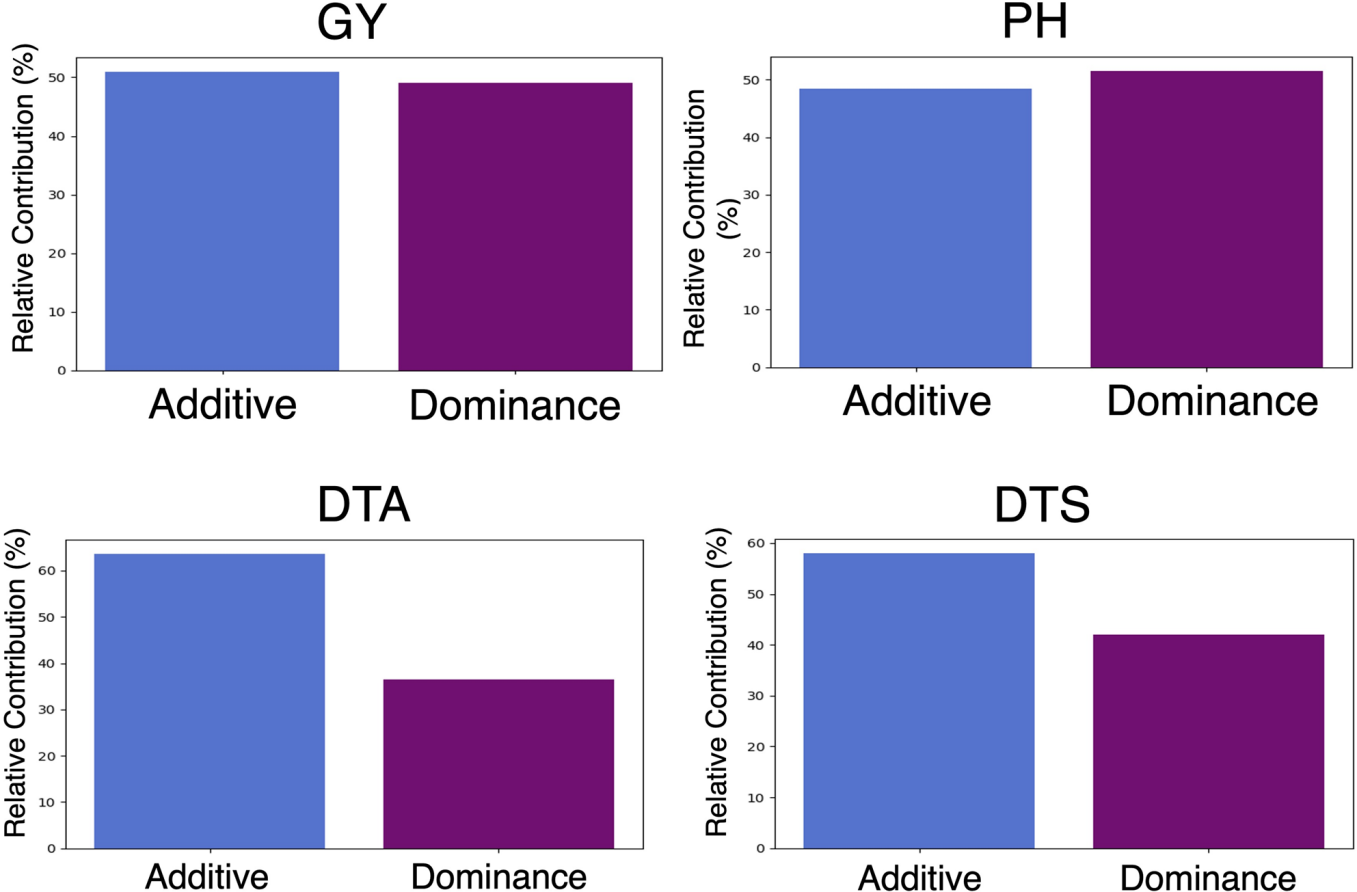
Relative contribution of additive and dominance SNP markers to model predictions for each of the four agronomic traits evaluated: Grain Yield, Plant Height, Days to Anthesis, and Days to Silking. The bar plot displays the proportion of the total absolute Shapley Additive Explanation (SHAP) values attributed to each effect type (additive in blue and dominance in purple), reflecting their relative influence on the model output

**Fig. 9:**
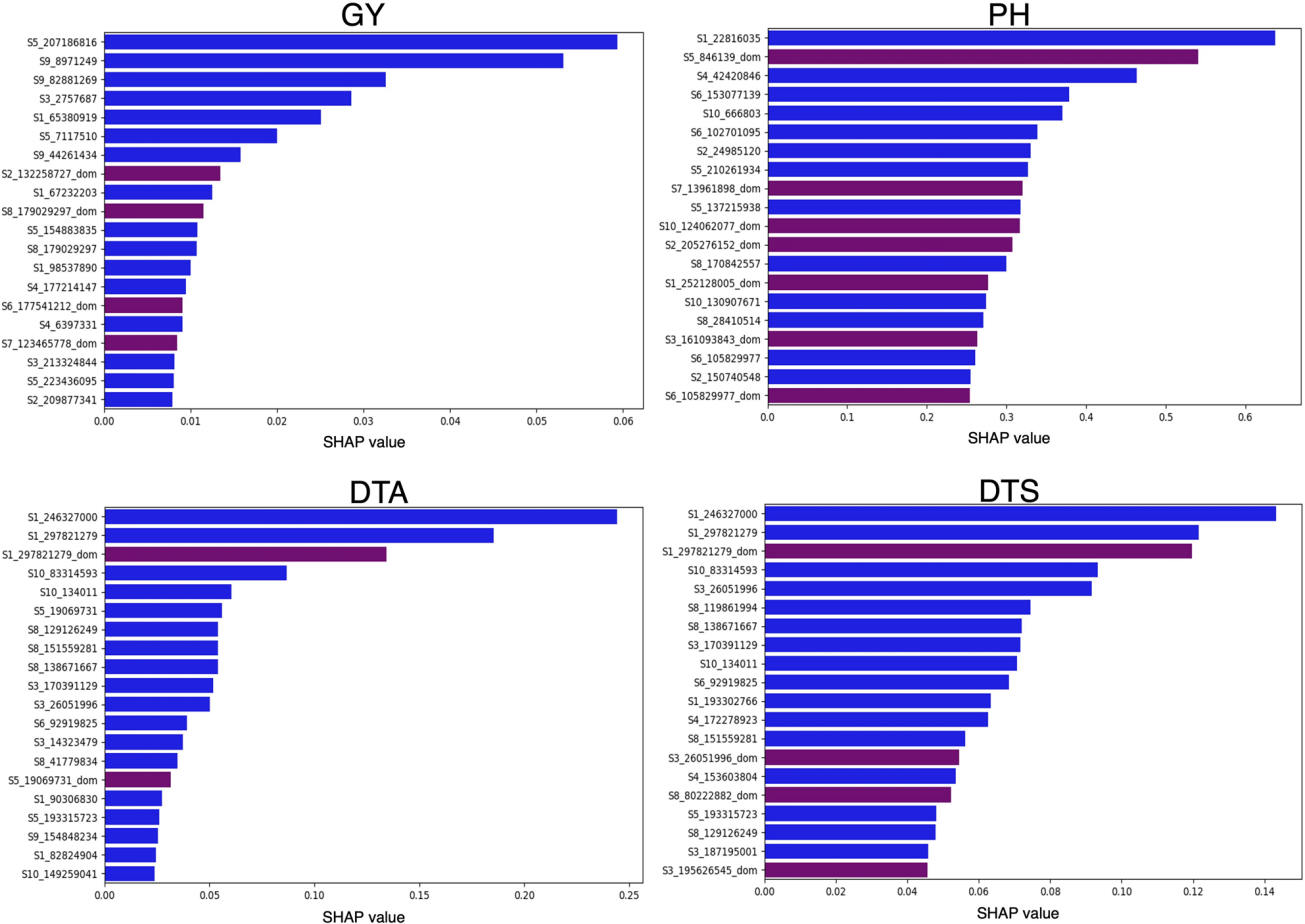
Shapley Additive Explanation (SHAP) summary plots showing the absolute contribution of the top 20 SNP markers for the four agronomic traits evaluated in this study. Each panel corresponds to one trait (Grain Yield, Plant Height, Days to Anthesis, and Days to Silking), with SNP markers ranked along the y-axis according to their relative importance in model predictions. The x-axis represents the magnitude of SHAP values, indicating each marker’s contribution to the model’s output. Blue-colored points represent the additive component of the 2NP matrix, while purple-colored points correspond to the dominance component of the 2NP matrix. This visualization illustrates how both additive and dominance effects contribute to trait prediction in the hybrid maize population

We conducted SHAP analysis to evaluate feature importance within the 2NPLGBM model. Specifically, we identified the top 20 variables derived from the 2NP matrix that contributed most significantly to phenotypic variation across traits. For Grain Yield, Days to Anthesis, and Days to Silking, the top-ranked variables were predominantly additive. In contrast, Plant Height exhibited a greater proportion of dominance variables, with seven of the top 20 features originating from the dominance component of the 2NP matrix (Fig. 8 & 9 ). These findings are consistent with the overall decomposition of genetic effect contributions obtained from the GBLUP model.

By aggregating SHAP values for additive and dominance variables from the 2NP matrix, we quantified the relative contributions of additive and dominance genetic effects to model performance. For Plant Height, dominance was the most significant contributor to phenotypic variation (Fig. 8), while Days to Anthesis showed the lowest relative dominance contribution (Fig. 8). The observed ratio of additive to dominance variables among the top-ranked features aligns with the trait-specific contributions of genetic effect types. Correspondingly, variance analysis using the GBLUP method indicated that the dominance genetic contribution in this population was lowest for Days to Anthesis (0.098) and Days to Silking (0.087), as shown in Table 2.

To assess the genetic contributions to the 2NPLGBM model, we examined interactions between SNP variables representative of additive and dominance effects. Generally, the results were inconclusive, but we observed that, unlike the GBLUP models, where the additive and dominant main effects mainly contribute to model performance (See Table 2), the main contributors to the 2NPLGBM model are the interaction effects. See Supplementary Material 2 (SFig1 and SFig2).

 .

## Discussion

### The 2NPLGBM model: a hybrid approach to genomic prediction

The integration of genomic selection into plant breeding schemes has been shaped by two primary modeling cultures: the data model culture and the algorithmic model culture [7]. Data models, most notably GBLUP [1, 2] and RR-BLUP [25, 26], have dominated for many years due to their simplicity, interpretability, and effective handling of additive genetic effects. In contrast, algorithmic models based on machine learning [26, 34–38] and deep learning [39, 41, 45, 47, 50] have emerged over the last decade due to their ability to utilize non-linear and complex relationships without making assumptions about the underlying genetic architecture. The expression of quantitative traits, however, is influenced by both additive and non-additive components, including dominance and epistasis [68]. In data models, these effects are often modeled via multi-kernel extensions, while algorithmic models tend to capture these effects intrinsically.

In this study, we provide a comprehensive assessment of a hybrid genomic prediction model, 2NPLGBM, across multiple traits, validation schemes, and years in hybrid maize. By integrating a genotype matrix grounded in quantitative genetic theory (2NP) with a gradient boosting algorithm (LGBM), we demonstrate how structured genomic information can enhance predictive performance and selection efficiency, offering new perspectives for selection in breeding. The 2NP matrix is a genotype representation obtained by concatenating additive and dominance matrix representations. The assumption of dependency between additive and dominance effects in the 2NPLGBM model more closely reflects reality, where these effects often interact to shape complex trait expression. This may account for the improved predictive performance and selection efficiency observed in this study, particularly for highly heritable traits, where dominance and interaction effects can contribute to phenotypic variance [17].

In addition, by incorporating additive and dominance features through the 2NP matrix in the machine learning method and employing Shapley Additive exPlanations (SHAP), we were able to dissect the relative contributions of additive, dominance, and their interactions to model predictions. Traits for which the 2NPLGBM outperformed classical methods and its machine learning counterpart (LGBM) tended to exhibit higher interaction contributions and higher heritability, although this pattern was not consistent across all validation schemes. This highlights an important implication: the 2NPLGBM model is mostly trait-dependent, with its greatest advantage emerging when non-additive (epistasis and other genetic interactions) effects play a major role in phenotypic variation. In such contexts, our model provides a robust framework for predicting total genetic values rather than just additive breeding values, thereby enabling more informed parental selection and combination design.

### Genomic predictive ability and selection efficiency

Previous research has shown that non-additive effects, including dominance and epistasis, play a modest yet trait-dependent role in plant breeding. Classical genomic prediction models [12, 13, 69] incorporated dominance effects but generally produced little to no improvement in predictive ability compared to additive-only models. Similarly, studies investigating genetic interactions [15, 17] reported modest or negligible gains when modeling non-additive effects, with the observed impact largely dependent on the genetic architecture of the trait and the population structure.

With the increasing adoption of machine learning in genomic selection, recent studies have explored transforming genomic data for ML- or DL-powered prediction, focusing on either dominance effects [70, 71] or epistatic interactions [72, 73]. Although these models have achieved modest improvements for traits influenced by non-additive effects, most were trained to capture only one type of genetic interaction at a time.

In contrast, the 2NPLGBM model introduces a structured genomic representation that simultaneously captures additive, dominance, and higher-order interactions (additive × additive, additive × dominance, and dominance × dominance). Evaluations in a maize population demonstrated that 2NPLGBM increased genomic predictive ability by over 5% in temporal validation schemes (LOYO and RW) and by more than 15% in tester-based validation schemes (Tester CV0 and Tester CV00), with the most significant gains observed for flowering traits (DTA and DTS). These improvements arise because the model directly integrates and exploits non-additive genetic signals, which are particularly relevant for traits where dominance and epistatic interactions contribute substantially to phenotypic variance. Importantly, 2NPLGBM achieved higher selection efficiency (and also matches LGBM for few scenarios that it did not outperform it) for flowering traits across all validation schemes except five-fold CV, indicating that non-linear models can detect subtle genetic signals that are important for selection decisions, even if their overall prediction metrics, such as accuracy or correlation, appear less favorable compared to linear models (the selection efficiency result is robust across different levels of selection see Supplementary Material 1(Sheet 10)). This has been demonstrated by several studies, which found that non-linear approaches may uncover complex genetic patterns or interactions that would otherwise be missed [74–78]. Therefore, incorporating these interaction components within the model may have enhanced model stability and indirectly support improved ranking performance. Its strength lies not only in modeling non-linear and dominance effects but also in delivering superior ranking performance, arguably the most critical metric in early-generation selection and resource allocation.

The overall contribution of non-additive effects to phenotypic variation remains trait-dependent [79]. For traits associated with hybrid vigor, models that explicitly capture these effects, such as 2NPLGBM, can yield superior predictive performance. Thus, aligning genomic prediction models with the underlying genetic architecture of the target trait is essential for maximizing total genetic gain.

From a practical breeding perspective, the 2NPLGBM model offers a robust alternative for predicting total genetic values, as it jointly models additive and non-additive effects in a unified framework. A breeding strategy guided by 2NPLGBM predictions could enhance the performance of commercial populations by enabling the prediction of cross-specific performance without direct phenotypic evaluation. Furthermore, through SHAP-based or other feature-importance analyses, breeders can quantify genetic contributions and uncover dominance and interaction effects that drive performance. This interpretability facilitates more informed parental selection decisions, accelerates test-cross hybrid development cycles, and ultimately enhances the efficiency and precision of genomic-assisted breeding pipelines.

A key consideration in applying genomic prediction models that include non-additive effects is their relevance to different breeding objectives. In parental line selection, where the goal is to identify suitable parents for subsequent breeding cycles [80], the primary target is breeding value, which reflects the additive genetic component transmitted to the next generation. In this context, models that explicitly estimate additive effects are generally more appropriate. By contrast, the approach proposed here is designed to capture total genetic value, including additive, dominance, and interaction effects, through the 2NP matrix representation combined with machine learning. This makes the method particularly suitable for applications such as test-cross hybrid performance prediction and cultivar evaluation [81], where non-additive effects contribute substantially to phenotypic performance. However, when the objective is parent selection for the next breeding cycle, the direct use of this model may be less appropriate unless additive and non-additive components can be explicitly separated.

### Limitation

Deep learning architectures (e.g., DNNs, CNNs) were not explored in this study due to time constraints and suboptimal preliminary results with CNNs. Future work should investigate the integration of 2NP matrices with deep neural architectures or ensemble frameworks combining 2NPLGBM with DL models. Another limitation is the increased feature dimensionality resulting from matrix concatenation, which in turn increases computational demand. Additionally, while SHAP enhances interpretability, it does not directly quantify causal effects and should be interpreted as indicative rather than definitive evidence of interaction architecture.

## Conclusions and future directions

In conclusion, this study proposes a method that demonstrates the value of integrating structured genotype matrices incorporating prior knowledge from quantitative genetic theory with non-linear machine learning algorithms to improve genomic prediction and selection outcomes in breeding. Future research should focus on:

1. Incorporating environmental covariates to improve model transferability across sites and years,
2. Developing multi-trait and multi-environment versions of 2NPLGBM to leverage correlated traits jointly, and.
3. Integrating multi-omics data (e.g., transcriptomic or epigenetic features) to capture additional biological layers of regulation.

Such extensions will help further bridge the gap between classical quantitative genetics and machine learning, advancing the predictive and explanatory power of machine learning in genomic selection.

## Supplementary Information

Below is the link to the electronic supplementary material.

Supplementary Material 1. Sheet 1: Hyperparameters tuned with Bayesian optimization for LightGBM modelsimplemented. Sheet 2: Prediction ability and selection efficiency of LOYO validation scheme Sheet 3: Prediction ability and selection efficiency of RW validation scheme Sheet 4: Prediction ability and selection efficiency of 5-Fold validation Sheet 5: Prediction ability and selection efficiency ofTester_CVO validation scheme Sheet 6: Prediction ability and selection efficiency of Tester_CVOO validation scheme Sheet 7: Variance Components and Heritability estimate for all traits Sheet 8: Number of testers per year Sheet 9: Number of Hybrids per testers Sheet 10:Selection efficiency at 5%, 10%, 20% and 30% level of selection

Supplementary Material 2.

## Data Availability

We obtained the G2F dataset from the committee of The Genomes to Fields 2024 Maize Genotype by Environment Prediction Competition, accessible on CyVerse under [https://doi.org/10.25739/78mn-4394](https:/doi.org/10.25739/78mn-4394). A GitHub repository containing the bash scripts, R scripts, and Python scripts used for phenotypic and genotypic analysis, as well as all genomic predictions, is available at [https://github.com/BrightGuru/2NP\_Matrix-for-Genomic-Prediction](https:/github.com/BrightGuru/2NP_Matrix-for-Genomic-Prediction) **.**.

## Abbreviations

GBLUP: Genomic best linear unbiased prediction
ML: Machine learning
PH: Plant Height
GY: Grain Yield
DTA: Days to Anthesis
DTS: Days to Silking
QTL: Quantitative Trait Loci
SNP: Single-nucleotide polymorphisms
MAS: Marker-assisted selection
RCBD: Randomized complete block design
PHG: Practical Haplotype Graph
BLUE: Best linear unbiased estimates
PDV: Proportion of dominance variation calculated as the dominance variance divided by the total genotypic variance
$\:{d}^{2}$: The proportion of dominance variation, calculated as the dominance variance divided by the total phenotypic variance
$\:{h}^{2}$: Narrow-sense heritability
$\:{H}^{2}$: Broad-sense heritability
d: Estimated degree of dominance
GBDT: Gradient-boosting decision tree
CV: Cross-validation
