# Supplementary material for: 2NPLGBM: a genomic model that merges the strengths of classical and machine learning methods in genomic prediction
Source: Plant Methods. 2026 May 28;22:50. doi: 10.1186/s13007-026-01545-2 (PMC13221760; doi:10.1186/s13007-026-01545-2)
Supplement: Supplementary file 2 — Supplementary Material 2. [file 13007_2026_1545_MOESM2_ESM.docx]

**SUPPLEMENTARY FIGURE**


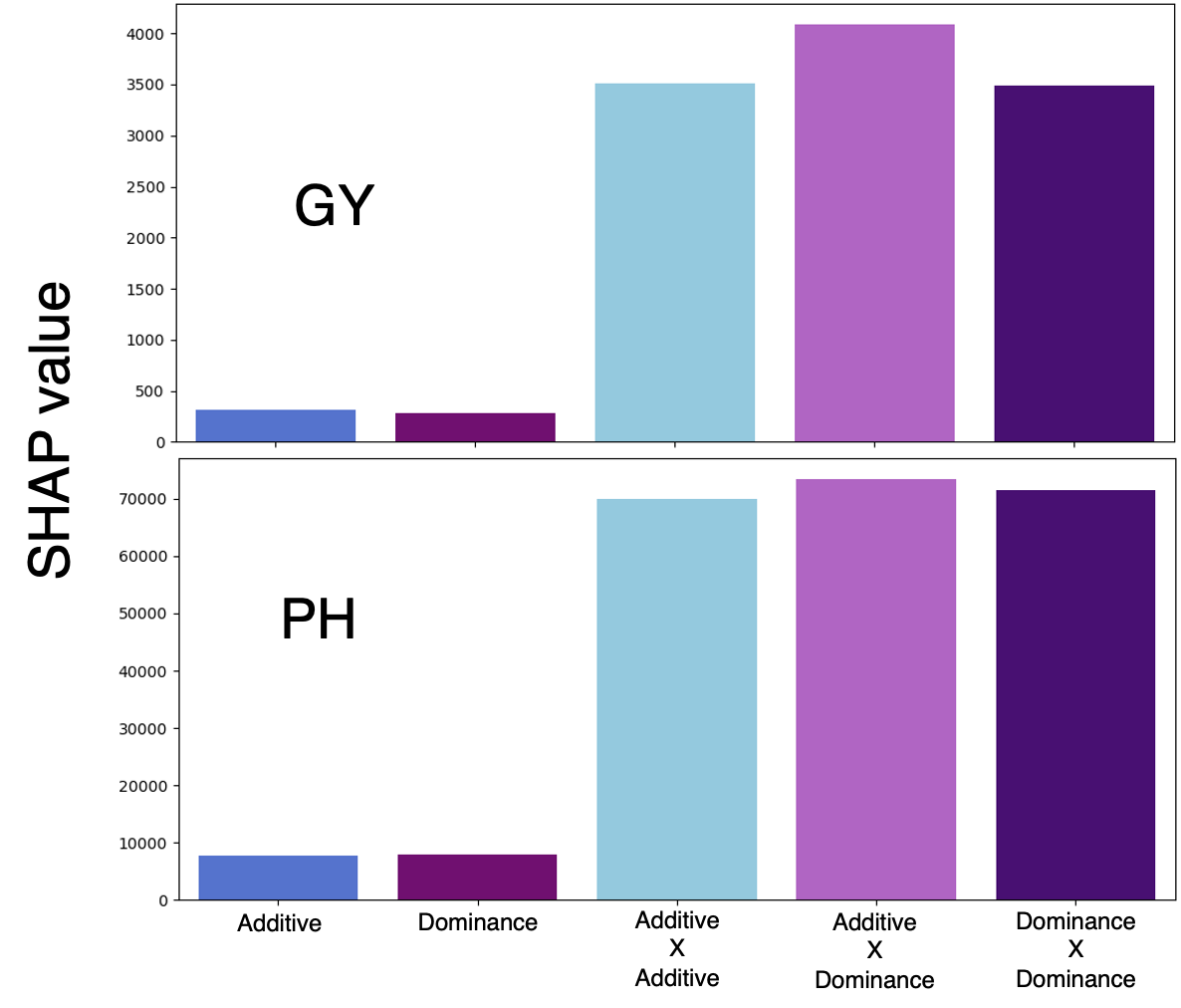


**SFig1.** Relative contribution of additive and dominance SNP markers to model predictions for Grain Yield and Plant Height. The bar plot displays the proportion of the total absolute Shapley Additive Explanation (SHAP) values attributed to each effect type (additive in blue, dominance in purple, Additive X Additive in light Blue, Additive X Dominance in light shade of purple and Dominance X Dominance in darker shade of purple), reflecting the relative influence of additive main, dominance main and interactive effects on 2NPLGBM model output.


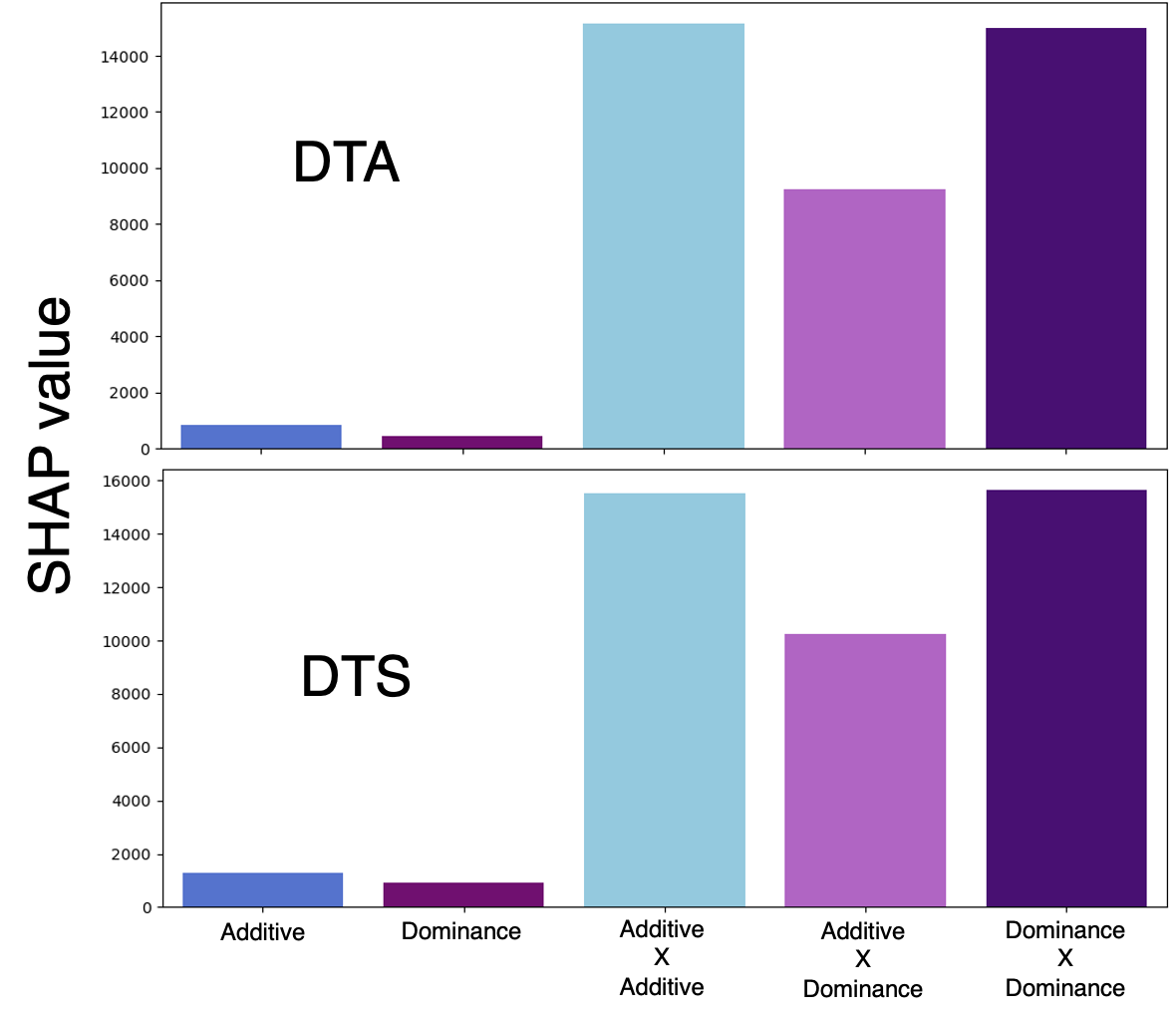


**SFig2.** SFig1. Relative contribution of additive and dominance SNP markers to model predictions for Days to Anthesis and Days to Silking. The bar plot displays the proportion of the total absolute Shapley Additive Explanation (SHAP) values attributed to each effect type (additive in blue, dominance in purple, Additive X Additive in light Blue, Additive X Dominance in light shade of purple and Dominance X Dominance in darker shade of purple), reflecting the relative influence of additive main, dominance main and interactive effects on 2NPLGBM model output.
